# Machine Learning for Detection of Safety Signals From Spontaneous Reporting System Data: Example of Nivolumab and Docetaxel

**DOI:** 10.3389/fphar.2020.602365

**Published:** 2021-01-14

**Authors:** Ji-Hwan Bae, Yeon-Hee Baek, Jeong-Eun Lee, Inmyung Song, Jee-Hyong Lee, Ju-Young Shin

**Affiliations:** ^1^ School of Pharmacy, Sungkyunkwan University, Suwon-si, South Korea; ^2^ Department of Health Administration, College of Nursing and Health, Kongju National University, Gongju-si, South Korea; ^3^ Department of Artificial Intelligence, Sungkyunkwan University, Suwon-si, South Korea; ^4^ Samsung Advanced Institute for Health Sciences and Technology, Sungkyunkwan University, Jongno-gu, South Korea

**Keywords:** disproportionality analysis, nivolumab, docetaxel, signal detection, adverse drug reaction, machine learning algorithms

## Abstract

**Introduction:** Various methods have been implemented to detect adverse drug reaction (ADR) signals. However, the applicability of machine learning methods has not yet been fully evaluated.

**Objective:** To evaluate the feasibility of machine learning algorithms in detecting ADR signals of nivolumab and docetaxel, new and old anticancer agents.

**Methods:** We conducted a safety surveillance study of nivolumab and docetaxel using the Korea national spontaneous reporting database from 2009 to 2018. We constructed a novel input dataset for each study drug comprised of known ADRs that were listed in the drug labels and unknown ADRs. Given the known ADRs, we trained machine learning algorithms and evaluated predictive performance in generating safety signals of machine learning algorithms (gradient boosting machine [GBM] and random forest [RF]) compared with traditional disproportionality analysis methods (reporting odds ratio [ROR] and information component [IC]) by using the area under the curve (AUC). Each method then was implemented to detect new safety signals from the unknown ADR datasets.

**Results:** Of all methods implemented, GBM achieved the best average predictive performance (AUC: 0.97 and 0.93 for nivolumab and docetaxel). The AUC achieved by each method was 0.95 and 0.92 (RF), 0.55 and 0.51 (ROR), and 0.49 and 0.48 (IC) for respective drug. GBM detected additional 24 and nine signals for nivolumab and 82 and 76 for docetaxel compared to ROR and IC, respectively, from the unknown ADR datasets.

**Conclusion:** Machine learning algorithm based on GBM performed better and detected more new ADR signals than traditional disproportionality analysis methods.

## Introduction

Pharmacovigilance based on spontaneous reporting systems (SRSs) have been implemented for the early detection of rare or unknown adverse drug reactions (ADR) that were undetected in clinical trials. Various data-mining techniques have been developed and successfully implemented for post-marketing drug safety surveillance using medical databases (Arnaud et al., 2017). Disproportionality analysis (DPA) methods such as reporting odds ratio (ROR) and information component (IC) were frequently used to analyze the strength of association between a drug and an adverse event from SRSs (Pham et al., 2019).

However, these methods simply calculate indices based on a 2 × 2 contingency table to determine safety signals (Bate et al., 1998; Bate and Evans, 2009; Raschi et al., 2019), ignoring the influence of confounders in the analysis, which may lead to biased and inaccurate signals (Van Puijenbroek et al., 2002; DuMouchel et al., 2013; Candore et al., 2015). Furthermore, these methods apply identical thresholds for determining safety signals to different databases, which may lead to the under-detection of adverse drug reactions (ADRs) (Matsushita et al., 2007; Harpaz et al., 2013). To overcome these limitations, a signal detection study was conducted by applying machine learning algorithm to SRS data; however, ROR and IC achieved better area under the curve (AUC) scores (ROR: 0.67, IC: 0.69) than machine learning algorithms (random forest [RF]: 0.52, Monte Carlo logistic regression: 0.58) (Pham et al., 2019). However, the study evaluated predictive performance without separating entire data into a training set and a test set, which limited the interpretation of the findings. There remains a gap in knowledge regarding the feasibility of machine learning algorithms to generate potential ADR signals from post-marketing surveillance data.

To fill that gap, we aimed to compare the predictive performance of machine learning algorithms in detecting safety signals of two anti-cancer agents, namely nivolumab and docetaxel, from SRS data with that of traditional DPA methods.

## Materials and Methods

### Study Scheme

**FIGURE 1:**
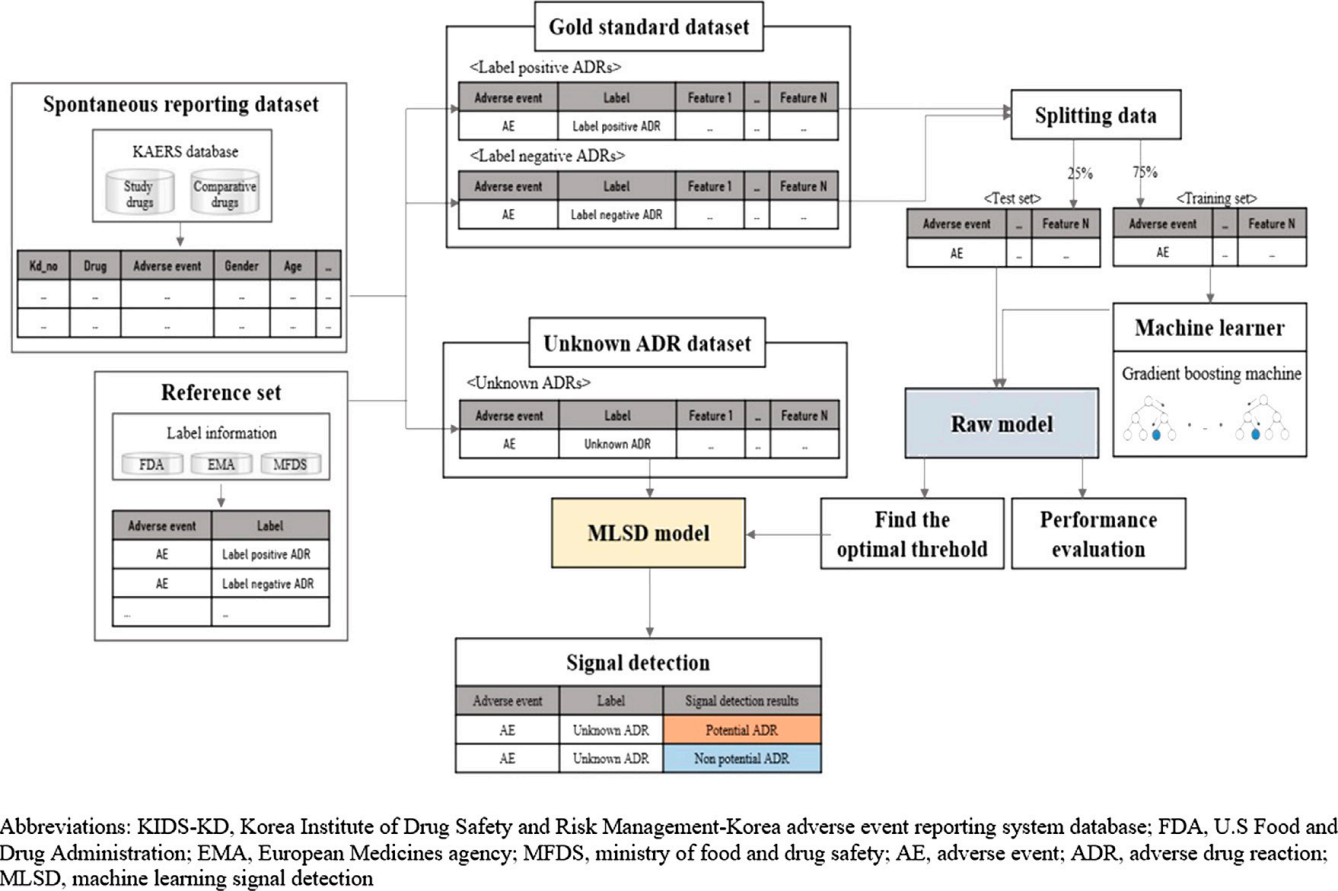
Overall scheme, performance evaluation, and application of machine learning signal detection.

Figure 1 presents the overall scheme of developing and implementing a machine learning signal detection (MLSD) model to predict potential safety signals. We used supervised learning algorithm, which represents a process of training the model with a subset of drug label data. We detected safety signals using the MLSD model via the following steps. First, all AEs associated with study drugs were extracted from SRS data and matched with label information based on the reference set. Then, we selected 23 features that could explain the occurrence of AEs and created novel input datasets that consisted of values for each AE. The novel input datasets were divided into gold standard datasets, which consisted of label-positive and label-negative ADRs, and unknown ADR datasets to implement machine learning algorithms. The algorithms were trained with 75% of gold standard data and the predictive performance of machine learning algorithms was evaluated with remaining 25% of the data. The algorithm that performed the best in the evaluation step was selected to develop the MLSD model. We then determined the optimal probability threshold that led to the best predictive performance of MLSD. By applying the optimal probability threshold, MLSD was implemented to detect signals from unknown ADRs, which were neither label-positive nor label-negative ADRs.

### Selection of Study Anticancer Agents

Nivolumab is one of the latest anticancer agents in the class of immune checkpoint inhibitors. The safety evaluation of the agent has become increasingly important with the rapid expansion of indications, but its safety profile is yet to be confirmed due to its relatively short history of use (Friedman et al., 2016; Martin-Liberal et al., 2017; Clarke et al., 2018). The number of AE reports and safety issues related to the liver, the lung, and the endocrine system has increased with the growing use of nivolumab; however, only a few post-marketing safety studies have been performed (Ji et al., 2019; Raschi et al., 2019). Therefore, we examined the safety profiles of nivolumab and six other agents, which were used as comparator drugs for nivolumab in clinical trials: dacarbazine, carboplatin, cisplatin, paclitaxel, docetaxel, and ipilimumab (Larkin et al., 2015; Rizvi et al., 2015; Weber et al., 2015; Horn et al. 2017).

Docetaxel, a traditional anticancer agent with proven safety profile, was selected as a second study drug to examine the validity of our study scheme. In contrast to nivolumab, docetaxel’s labels list updated and appropriate safety information, because many post-marketing safety studies have already been conducted (United States Food and Drug Administration, 2019). We selected eight comparator drugs for docetaxel based on the same criteria as for nivolumab: mitomycin, vinblastine, fluorouracil, doxorubicin, cyclophosphamide, vinorelbine, cisplatin, and mitoxantrone (Nabholtz et al., 1997; Fossella et al., 2000, Fossella et al., 2003; Tannock et al., 2004; Martin et al., 2005; Vermorken et al., 2007; Jones et al., 2009).

### Data Source

We used the Korea Institute of Drug Safety and Risk Management-Korea adverse event reporting system database (KIDS-KD) from 2009 to 2018. A computerized AE reporting system was established in Korea in 2012 to effectively manage AE reports and subsequently incorporated all spontaneous AE reports filed since 1988. The KIDS-KD includes information on the general characteristics of the patients, suspected drug code, ADR code, serious adverse event case, report type, reporter, and reporting institution. We extracted AE reports of nivolumab and its comparator drugs from January 2015 to December 2018, and for docetaxel and its comparator drugs from January 2009 to December 2018. Drugs were coded according to the World Health Organization’s anatomical therapeutic chemical classification system (ATC). Suspected AEs were coded by the preferred term, which is one of four classification levels of the World Health Organization-Adverse Reactions Terminology (WHO-ART) version 092 (World Health Organization-Uppsala Monitoring Centre, 2017).

### Constructing Novel Input Datasets

To implement machine learning algorithms for signal detection, we constructed the novel input datasets for study drugs that included label information and statistical, organ-specific, and covariate feature data specifically selected for machine learning. Machine learning algorithms are designed to accept novel input data and produce the probability that a specific AE is a potential ADR signal.

#### Labeled Data

Labeled data is comprised of gold standard data and unknown ADR data. The gold standard represents if each AE associated with a drug from SRS data is actually listed in the drug label and is used as the orientation for training and testing machine learning algorithm. In contrast, an unknown ADR refers to an identified AE that may be linked with the drug. Each algorithm used unknown ADR dataset to detect the potential ADR signals for study drugs.

To construct labeled data, a reference set was first created by extracting labeling information for all study drugs from the Korea Ministry of Food and Drug Safety, the United States Food and Drug Administration, and the European Medicines Agency. We created labeled data by identifying AEs from the KIDS-KD for each study drug and designating AEs into three ADR categories based on the reference data: label-positive, label-negative, and unknown ADRs. Label-positive ADRs were defined as the AEs that were listed in the labels of the study drug. Label-negative ADRs were defined as the AEs that were not listed in the labels of the study drug and other drugs in the same therapeutic class. These two categories of label-positive and label-negative ADRs constituted gold standard data whereas unknown ADR data denotes drug-AE pairs that were neither label-positive nor label-negative ADRs (Wahab et al., 2013; Harpaz et al., 2014; Hoang et al., 2018).

We assigned the drugs that shared the first five codes of ATC and the same mechanism of action with a study drug to the same therapeutic class with the study drug. For nivolumab, we selected pembrolizumab, atezolizumab, durvalumab, and avelumab as drugs of the same class, which are coded as L01XC and inhibit programmed cell death 1 (PD-1)/PD-L1. For docetaxel, we determined paclitaxel, and cabazitaxel to be in the same class, which are coded as L01CD and inhibit the process of cell division.

#### Feature Data

Feature data represents quantifiable properties and characteristics of AEs, which can be used to improve the performance of machine learning algorithms. The quality of features in a dataset has a key impact on the integrity of the analysis.

To enhance applicability of our model to other spontaneous reporting system databases, we considered variables related to the least required information that must be included in spontaneous reporting such as suspicious drug information, AE information, patient information, reporting information. Among the variables, we selected 22 features used in previous signal detection studies (Soukavong et al., 2016; Ran et al., 2019). Features were comprised of statistical and covariate features. We selected four statistical features (a, b, c, d) that have been used in traditional signal detection methods. Feature a is the number of reports of a specific AE associated with a particular study drug. Feature b is the number of reports of other AEs related with the study drug. Features c and d are the number of reports of a specific AE and other AEs, respectively, for the comparators (Table 1).

**TABLE 1:** Statistical features contained in a 2 × 2 contingency table.

Number of reports	Specific adverse event	Other adverse events
Study anticancer agents	Feature a	Feature b
Comparators	Feature c	Feature d

Covariate features represent the confounding factors that may affect signal detection results and included the patient’s sex, age, serious adverse events, report type, reporter’s occupation, and reporting institution. Age was divided into three groups based on groupings used in previous post-marketing surveillance studies; 0–17, 18–64, and 65 years of age and older (Ji et al., 2019; Raschi et al., 2019). Report types included AEs reported to the SRS, post-marketing surveillance study report, and the literature. There were five reporter occupation categories (physician, pharmacist, nurse, other health professional, and consumer) and three reporting institution types (regional pharmacovigilance center, medical institution, and drug manufacturer). The frequency of AE reports was calculated by each feature category.

As ADRs are manifested in specific organs, we also considered organ-specific features of AEs associated with study drug. Organ-specific features were grouped into system organ classes (SOCs) according to the WHO-ART version 092. SOCs are groupings of medical events by etiology (e.g., infections and infestations), manifestation site (e.g., gastrointestinal disorders), or purpose (e.g., surgical and medical procedures). All 23 features used in our study are listed and described in Supplementary Table S1.

### Statistical Analysis

#### Algorithms for Signal Detection

For signal detection, we used the gradient boosting machine (GBM) and random forest (RF), which are widely used machine learning algorithms for classification, and then compared the results with those obtained from two traditional DPA methods: ROR and IC.

##### GBM

Our MLSD model was developed by using GBM algorithm (Shai and Shai, 2014), which is a learning method that corrects the errors of its predecessor while learning weak learners sequentially and has a proven outstanding performance in classification (Liu et al., 2017; Hoang et al., 2018; Rahimian et al., 2018). Boosting is a generally used method to boost the accuracy of any learning algorithm by fitting a series of models and then combining these models into an ensemble, which performs better than any single model. GBM algorithm starts with fitting data with a simple decision tree, which in general performs slightly better than random guessing. The GBM algorithm proceeds to train another decision tree by using gradient descent of the loss function in order to reduce errors in the previous tree. This sequential process continues iteratively until it produces a model that fits the training set with minimal errors. Through this process, GBM optimizes criteria that can be applied to classify drug-AE pairs as either label-positive ADRs or label-negative ADRs.

##### RF

The RF is another ensemble method that combines multiple decision trees by bagging, which is a general method of aggregating learners trained by bootstrap samples (Shai and Shai, 2014). Each decision tree is at once trained by using bootstrap samples and their features, all of which are randomly drawn from the original training set. This makes each decision tree create independent criteria to classify drug-AE pairs as either label-positive ADRs or label-negative ADRs. Then, RF aggregates all decision trees and determines the classes of drug-AE pairs by a majority vote, which makes RF have generalizability to other datasets.

##### DPA

ROR and IC are traditional DPA methods, frequently used to detect ADR signals. ROR represents the odds of a specific AE occurring in the patient exposed to a specific drug divided by the odds of occurrence of an AE specific to comparative drugs. We calculated ROR for each AE and determined that it is a signal, based on the following parameters; We used a multivariable logistic regression model to estimate ROR and 95% credibility interval (CI) adjusted for sex, age, cases of serious adverse event, report type, reporter’s occupation, reporting institution, and SOC. Safety signals were considered significant when the lower limits of the corresponding CI for the adjusted ROR are ≥1 (Harpaz et al., 2013). IC is a logarithmic metric of the value calculated by dividing the probability of drug use and a specific AE by the product of the probability of drug use and probability of a specific AE occurring when drug use and the occurrence of the specific AE are independent. The criterion for signal detection was an IC025 of >0 where IC025 is the lower end of a 95% CI for the IC (Bate et al., 1998).

#### Training Machine Learning Algorithms

The Synthetic Minority Over-sampling Technique (SMOTE) was applied to the gold standard datasets to adjust for an imbalance in the distribution of labels in the dataset between label-positive and label-negative ADRs. The dataset was then randomly divided into a training set and a test set (75% and 25%, respectively). We fitted GBM and RF with hyperparameters tuned on the training set by using a stratified five-fold cross-validation, which is a resampling technique to evaluate machine learning models on a limited data sample by dividing data into five subsets (Table 2). In the first iteration, the first fold is used as test set and the remaining groups serve as training sets. This process was repeated until each fold of the five folds was used as the test set.

**TABLE 2:** Hyperparameters defined for machine learning signal detection in the nivolumab and docetaxel training sets.

Parameter	Dataset	
	Nivolumab	Docetaxel
Eta	0.01	0.01
Num_boost_rounds	100	100
Max_depth	4	6
Min_child_weight	1	1
Colsample_bytree	0.4	0.7
Gamma	0	0
Random_state	200	200

#### Evaluating Signal Detection Algorithms

To evaluate the predictive performance of GBM and RF, which are based on supervised learning with a subset (training set) of gold standard data, we used the remainder of the data (test set). However, the performance of ROR and IC was assessed by using entire gold standard data. Performance was measured as the AUC of the receiver operating characteristic curve obtained by plotting the true positive rate against the false positive rate at various threshold settings. The true positive rate is also known as sensitivity and false positive rate is calculated as 1 - specificity.

Then, we selected the machine learning algorithm with best performance and determined the optimal probability threshold that led to the best predictive performance of the selected algorithm to develop MLSD. To determine the optimal probability threshold, we calculated the AUC score on a given threshold probability by varying it from 0.0 to 1.0 with an increment of 0.01. If several threshold values achieved the same AUC score, we selected the highest value as the optimal threshold. The values of accuracy, sensitivity, specificity, positive predictive value, and negative predictive value on the optimal threshold were calculated for each study drug. Accuracy was the proportion of true results among the total number of cases examined. Sensitivity was the proportion of label-positive ADRs in gold standard data that were predicted as signals by MLSD. Specificity was the proportion of label-negative ADRs in gold standard data that were predicted as non-signals by the model. A positive predictive value was the probability of predicting ADR signals as label-positive ADRs. Conversely, a negative predictive value was the probability of predicting non-signals as label-negative ADRs.

#### Signal Detection

Based on the verified optimal probability threshold in performance evaluation, machine learning algorithms were implemented to determine whether each AE in the unknown ADR dataset was a potential ADR signal. We compared the signals obtained by MLSD with those by DPA, by presenting the signals detected by each algorithm on a scatter plot. The y-axis and the x-axis indicate the optimal probability threshold of MLSD and each DPA, respectively. The AEs detected as signals by both MLSD and DPA were presented in the first quadrant; those detected as non-signals by both methods were in the third quadrant; signals detected by either method were in the second and fourth quadrants.

All statistical analyses were performed using Python software version 3.7.5 (Python Software Foundation, Wilmington, DE, United States), SAS software version 9.4 (SAS Institute Inc., Cary, NC, United States), and Microsoft Office 365 ProPlus (Microsoft Corp., Redmond, WA, United States). This study was approved by the institutional review board of Sungkyunkwan University (2019-04-020-001), which waived informed consent, as only deidentified data were used in this study.

## Results

### Characteristics of Novel Input Datasets

**FIGURE 2:**
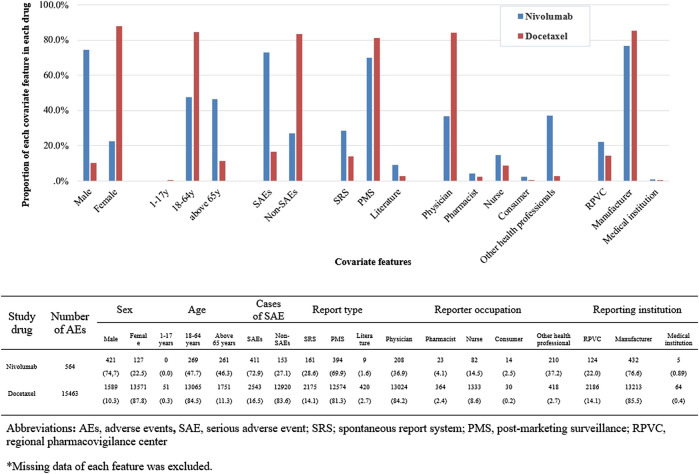
Comparison of covariate features between nivolumab and docetaxel input datasets.

**FIGURE 3:**
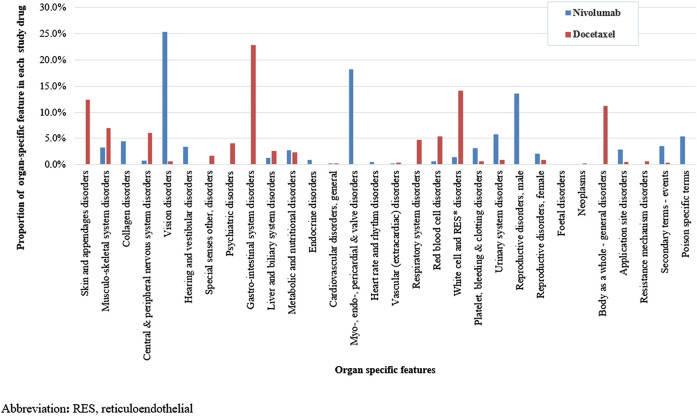
Comparison of organ-specific features between nivolumab and docetaxel input datasets.

We identified a total of 136 and 485 suspected AEs for nivolumab and docetaxel, respectively, from the novel input datasets (Table 3). Among the 136 AEs for nivolumab, 51% were label-positive, 11% label-negative, and 38% unknown ADRs. Among the 485 AEs for docetaxel, 55% were label-positive, 15% label-negative, and 30% unknown ADRs. Male patients reported most of the AEs for nivolumab (74.7%), while females did most of AEs for docetaxel (87.8%) (Figure 2). Most of AEs (72.9%) for nivolumab but only 16.5% of AEs for docetaxel were serious. While physicians reported most (84.2%) of AE cases for docetaxel, physicians (36.9%) and other health professionals (37.2%) reported nearly the same number of AEs for nivolumab. AEs for nivolumab were most commonly manifested as vision disorders, myo-, endo-, pericardial, and valve disorders, and general and male reproductive disorders and those for docetaxel as gastrointestinal system disorders, white cell and reticuloendothelial disorders, and skin and appendages disorders (Figure 3).

**TABLE 3:** Distribution of labels for two input data.

Study drugs	Suspected ADRs	Gold standard		Unknown ADRs^‡^
		Label-positive ADRs*	Label-negative ADRs^†^	
Nivolumab	136 (100%)	70 (51%)	15 (11%)	51 (38%)
Docetaxel	486 (100%)	267 (55%)	71 (15%)	148 (30%)

ADR, adverse drug reaction.

* Label-positive ADRs are defined as the adverse events (AEs) of respective drug that were listed in the labels of the Ministry of Food and Drug Safety, U. S. Food and Drug Administration, and European Medicines Agency.

† Label-negative ADRs are defined as the AEs not listed in the labels of the target drug and any other drugs in the same therapeutic class and considered unlikely to be ADR signals by experts.

‡ Unknown ADRs are drug-AE pairs neither label-positive ADRs nor label-negative ADRs.

### Developing an MLSD Model

**FIGURE 4:**
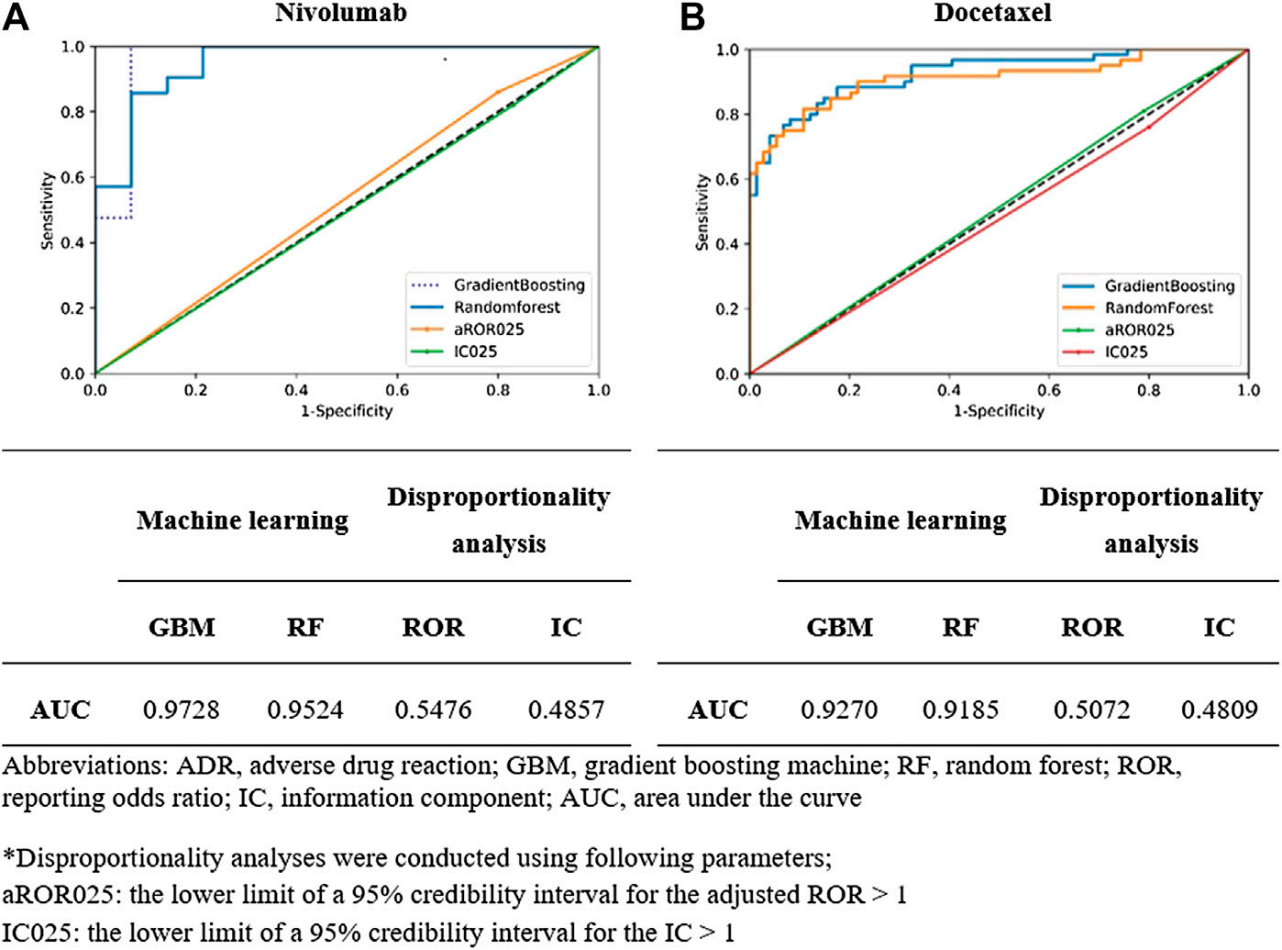
**(A)** The prediction performance of signal detection for classifying adverse events for nivolumab in the gold standard dataset using two different machine learning algorithms and two different disproportionality analysis methods. **(B)** The prediction performance of signal detection for classifying adverse events for docetaxel in the gold standard dataset using two different machine learning algorithms and two different disproportionality analysis methods.

**FIGURE 5:**
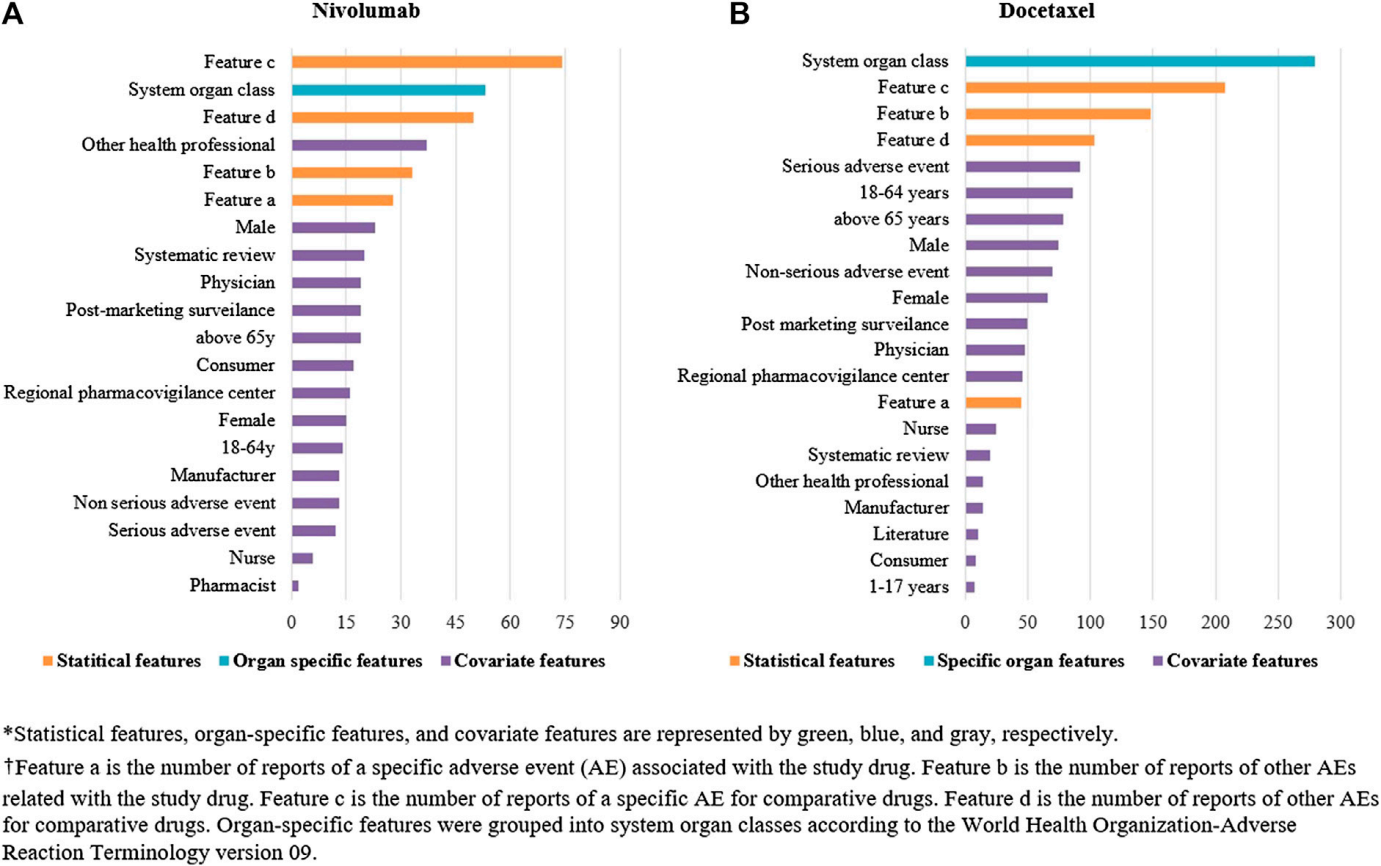
**(A)** The relative importance of features in detecting signals in nivolumab by machine learning. **(B)** The relative importance of features in detecting signals in nivolumab by machine learning.

**FIGURE 6:**
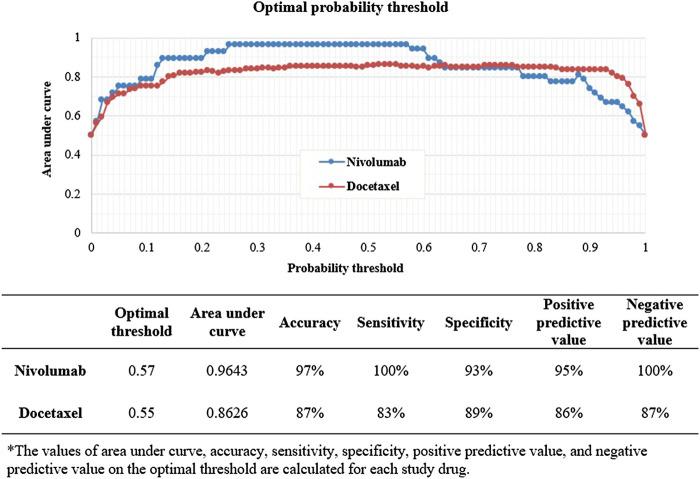
The prediction performance of machine learning signal detection on the optimal probability threshold.

Among the four algorithms implemented, GBM achieved the highest predictive performance in detecting ADR signals from the test datasets (AUC scores: 0.9728 and 0.9270 for nivolumab and docetaxel, respectively; Figures 4A,B. Feature b, feature c, feature d, SOC, and reporting by other health professionals had the greatest influence on signal detection for nivolumab in the probability calculation using GBM (Figure 5A). The most prominent features for docetaxel were SOC, feature b, feature c, feature d, and the number of serious AEs (Figure 5B). GBM yielded the highest AUC score of 0.9643 for nivolumab and 0.8626 for docetaxel at the optimal probability threshold value of 0.57 and 0.55, respectively (Figure 6).

### Predicting Signals From the Unknown ADR Dataset

**FIGURE 7:**
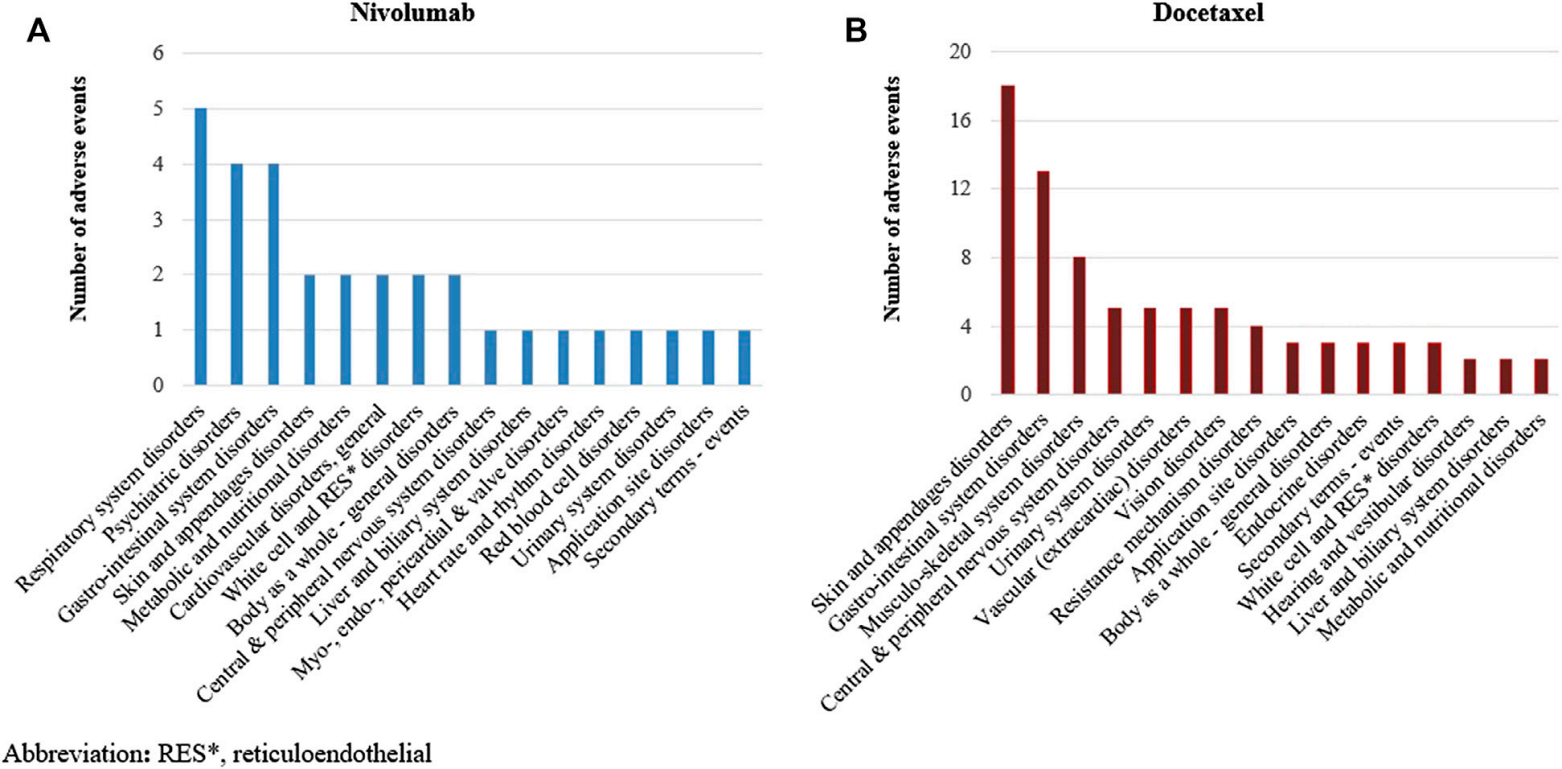
**(A)** Distribution of signals detected by machine learning signal detection by manifestation site in nivolumab. **(B)** Distribution of signals detected by machine learning signal detection by manifestation site in docetaxel.

**FIGURE 8:**
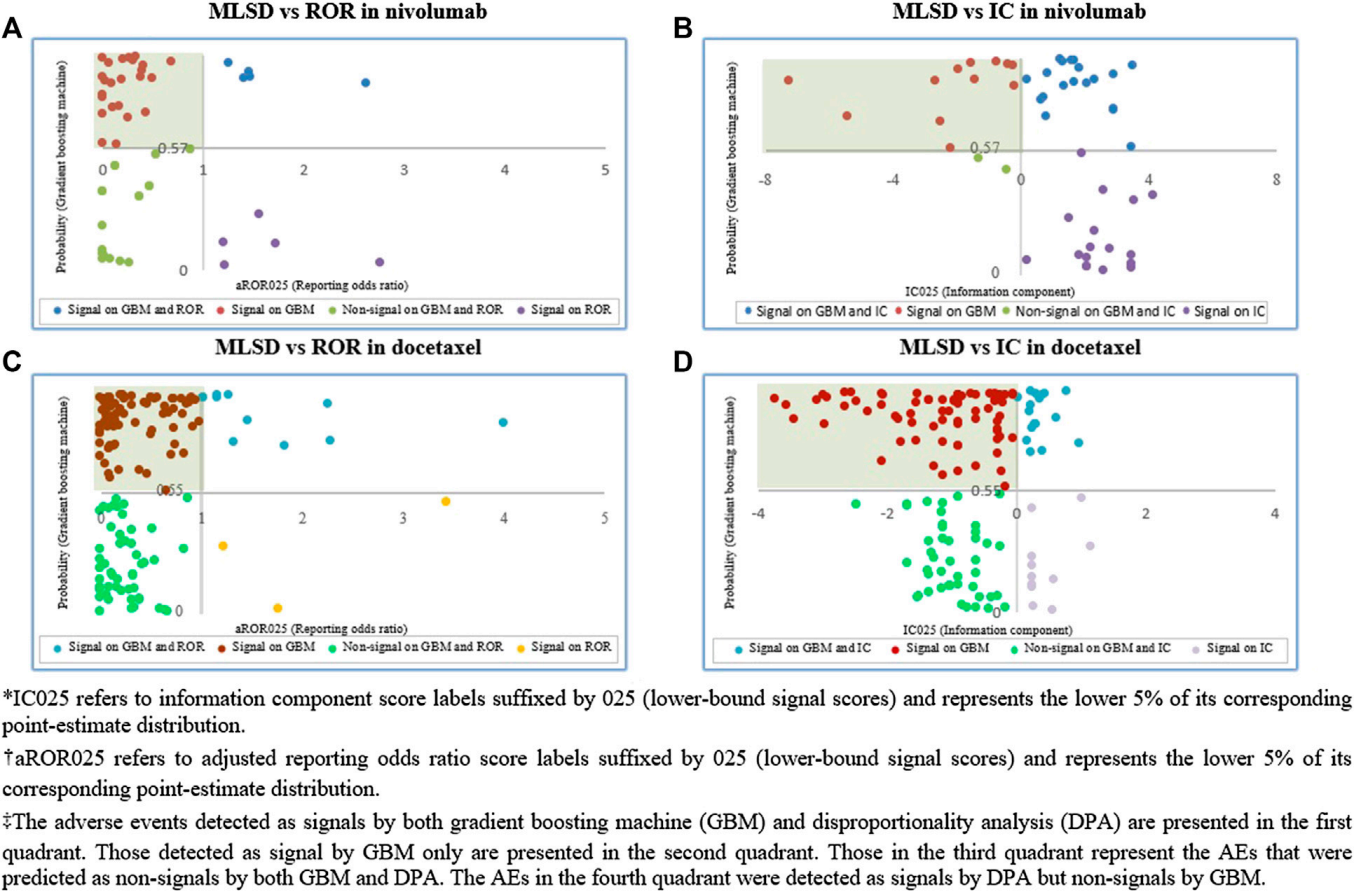
**(A)** Comparison of signals detected by machine learning signal detection (MLSD) and reporting odds ratio (ROR) in nivolumab. **(B)** Comparison of signals detected by MLSD and information component (IC) in nivolumab. **(C)** Comparison of signals detected by MLSD and ROR in docetaxel. **(D)** Comparison of signals detected by MLSD and IC in docetaxel.

Among the 51 unknown ADRs identified for nivolumab, MLSD generated 31 (61%) potential signals, which were mainly manifested as respiratory system disorders, psychiatric disorders, and gastrointestinal system disorders (Table 4; Figure 7A). Among the 148 unknown ADRs identified for docetaxel, MLSD detected 93 (63%) potential signals, which were mostly manifested as skin and appendages disorders, gastrointestinal system disorders, and musculo-skeletal system disorders (Table 4; Figure 7B). The signals in the second quadrant of each scatter plot in Figure 8 represent potential ADR signals detected by MLSD but undetected by DPA. MLSD detected additional novel 24 and nine signals (Figures 8A,B) for nivolumab and 82 and 76 signals (Figures 8C,D) for docetaxel, compared with ROR and IC, respectively.

**TABLE 4:** The number and percentage of signals detected by machine learning signal detection and traditional methods.

Study drug	Number of unknown ADRs	Number of signals		
		MLSD	ROR	IC
Nivolumab	51 (100%)	31 (61%)	9 (18%)	37 (73%)
Docetaxel	148 (100%)	93 (63%)	14 (9.6%)	28 (19%)

ADR, adverse drug reaction; MLSD, machine learning signal detection; ROR, reporting odds ratio; IC, information component.

## Discussion

Our signal detection study based on large SRS data was designed to evaluate the predictive performance of machine learning algorithm and the ability to detect unknown safety signals, compared to traditional DPA methods. Our novel machine learning algorithm, MLSD, outperformed ROR and IC in predicting signals for both nivolumab and docetaxel and detected a greater number of unknown ADR signals than ROR and IC. Although the traditional DPA methods perform well in some cases, the accuracy of signal detection may be limited by noise (resulting in false positives) and missing of some important relationships (resulting in false negatives). These limitations are attributable to the simplicity with which these methods calculate signal indices and the application of a uniform threshold regardless of the different characteristics of databases.

To overcome these limitations, we developed MLSD, a machine learning framework for signal detection based on GBM. The first key step in the process was the creation of the novel input data set by collating statistical, organ-specific, and covariate features. Using the comprehensive set of variables and drawing on subtle differences in features between label-positive and label-negative drug-AE pairs, MLSD calculated signal indices. Among the algorithms examined, MLSD attained the highest accuracy in detecting true ADRs while filtering out ADRs with spurious association. MLSD performed remarkably well in signal detection, considering that the accuracy of the gold standard diagnostic tool of prostate cancer and breast cancer was only 0.8 in previous studies (Martin et al., 2004; Pisano et al., 2005).

MLSD’s outperformance can be explained by its several underlying strengths. First, MLSD utilized a large number of features when calculating signal indices. A signal detection study, using the Food and Drug Administration’s adverse event reporting system (FAERS) database, showed that adjustment for confounders enhanced the predictive performance of logistic regression models (Harpaz et al., 2013). This indirectly explains the good performance of our MLSD. Second, unlike DPA methods, MLSD can calculate the effects of each feature on signal prediction differently depending on the datasets. In our analyses, the magnitude of the influence of each feature and the optimal probability threshold were different between nivolumab and docetaxel. MLSD may have improved its predictive performance by applying different weightings to feature variables depending on the characteristics of each dataset. Lastly, MLSD can generate safety signals with enhanced accuracy by fitting a model to different data and modifying the optimal probability threshold accordingly. In our study, the optimal probability thresholds which led to the highest AUC score were 0.57 and 0.55 for the nivolumab and docetaxel test sets, respectively. The difference suggests that determining the optimal probability threshold fitted to respective input data sets is important to elevate the predictive performance of machine learning algorithms.

Consistent with our finding that MLSD performed better than traditional DPA methods, a signal detection study using the Australian medication dispensing data demonstrated that GBM achieved the best performance in detecting ADR signals among the six widely used machine learning methods (Hoang et al., 2018). Several other empirical studies confirmed the superiority of the gradient boosting classifier over the RF classifier (Caruana and Niculescu-Mizil, 2006; Caruana et al., 2008). Consistent results were found in the additional analyses by using different parameters (Supplementary Figure S1).

Our MLSD detected new potential ADRs that were undetectable by DPA methods. MLSD detected 24 and nine more signals for nivolumab than ROR and IC, respectively. Among these signals, seven including cholecystitis and bronchiolitis were detected by MLSD only. A case-series study showed that immune checkpoint inhibitors including nivolumab can result in a clinical condition similar to typical acute cholecystitis, which should be managed as such, in a minority of patients (Abu-Sbeih, et al., 2019). Bronchiolitis was also reported as a pulmonary complication of nivolumab by several clinical studies and one meta-analysis study (Gounant et al., 2016; Cadranel et al., 2019; Verzoni et al., 2019). The results of these studies indicated that nivolumab may have caused organizing bronchiolitis syndrome and deaths. Combined with these previous studies, our findings suggested that MLSD can produce potential ADR signals of clinical significance that cannot be detected by DPA methods.

MLSD can be used as a potential strategy to detect rare AEs of newly marketed drugs. It is challenging to identify rare AEs of a drug at the time of its approval when only limited safety data are available. Therefore, it is important to detect rare AEs through post-marketing surveillance studies. However, traditional methods are not sensitive enough to detect rare AEs, resulting in false negatives (Sardella and Lungu, 2019). Hence, several regulatory authorities recommend that DPAs be complemented with other appropriate methods to identify rare safety signals (Kimura et al., 2011; Jos, 2015; Hesha, 2018). To assess the robustness of our MLSD model, we conducted a sensitivity analysis by adding a feature indicating whether an AE is rare or not. The result confirmed the robust predictive performance of MLSD, suggesting that MLSD detect potential signals of rare AEs with few errors (Supplementary Table S2). Nonetheless, it should be noted that MLSD cannot replace other forms of reporting, particularly for rare events and designated medical events (DME). For the signal detection of such events, the absolute number of reports can be more relevant than DPA methods and therefore, quantitative approaches should be used in combination with the results of qualitative methods.

Our study has several strengths. First, we used a nationwide spontaneous reporting database for all anti-cancer agents from 2009 to 2018. Second, this study is the first to apply GBM to detect safety signals from SRS data. Our MLSD model based on GBM performed the best in signal detection among the four algorithms implemented. Third, we empirically evaluated the predictive performance of MLSD and the ability to detect unknown signals compared to traditional DPA methods, to verify the feasibility of this novel approach in post-marketing safety surveillance. Finally, safety information on the label of nivolumab remained unchanged throughout the study period, which therefore circumvented publication bias.

Our study also has some limitations. First, passive surveillance data are inherently subject to potential selection bias and underestimation of AE reports (Alvarez et al., 1998). Another potential bias may arise from the selection of anti-cancer agents, because the generally poor prognosis of cancer patients may influence the frequency of AE reports. To minimize the bias, we used two study drugs that have differential quantities of accumulated safety information; nivolumab with less information due to a short duration of use and docetaxel with more information due to a long duration of use. Second, our findings can be affected when WHO-ART, used for defining AEs in KIDS-KD, is replaced by medical dictionary for regulatory activities (MedDRA), widely used in other SRS databases. However, as one WHO-ART term matches multiple MedDRA terms, we judged selecting one particular MedDRA term may introduce additional bias in our case study (Elliot, 2002). Third, “impure” gold standard can adversely affect the assessment of performance (Hauben et al., 2016). Assigning reference events to wrong classes may introduce a bias to performance metrics. However, we attempted to minimize this misclassification by reviewing labeling information from three regulatory authorities. We also applied the SMOTE to adjust class imbalance, another source of bias, and to minimize its influence. Fourth, our findings may have limited generalizability, because the performance of MLSD greatly depends on the features of training data (Hoang et al., 2018). To enhance the applicability of MLSD to other databases, we selected features from the most commonly reported information by other SRS databases. In addition, we applied stratified five-fold validation and hyperparameter tuning techniques to prevent the machine learning algorithms from over-fitting to training datasets. These techniques reduced the dependency of MLSD on the features of training sets.

In conclusion, MLSD, our novel machine learning algorithm based on GBM, achieved a better performance and detected more new ADR signals than traditional DPA methods. Our MLSD model could provide regulatory authorities with new and reliable insights into generating drug safety information from large SRS data. However, our machine learning algorithm is a preliminary attempt to introduce machine learning in the signal detection area and has not yet to be validated by any regulatory authorities. In addition, we assessed the performance of MLSD in two drugs only. Further post-marketing surveillance studies applying MLSD to other drugs may confirm the generalizability of our findings.

## Data Availability

The data analyzed in this study is subject to the following licenses/restrictions: Our study used Korea Institute of Drug Safety & Risk Management- Korea adverse event reporting system database (KIDS-KD) (Data number: 1905A0022). KIDS forbids the transfer, rent, or sale of the database to any third party other than the researcher, who obtained the approval for the provided database. Requests to access these datasets should be directed to Korea Institute of Drug Safety & Risk Management; Official website of KIDS: http://open.drugsafe.or.kr/; Contact information of data access committee: +82-2-2172-6700.
