# Supplementary material for: Machine Learning for Detection of Safety Signals From Spontaneous Reporting System Data: Example of Nivolumab and Docetaxel
Source: Front Pharmacol. 2021 Jan 14;11:602365. doi: 10.3389/fphar.2020.602365 (PMC7898680; doi:10.3389/fphar.2020.602365)
Supplement: Supplementary file 1 [file table1.docx]

**Table S1**. Description of 23 features used to classify adverse drug relactions

| **Feature** | **Description** |
| --- | --- |
| Statistical features | |
| Feature a | The number of reports of a specific AE for a particular study drug |
| Feature b | The number of reports of other AEs for the study drug |
| Feature c | The number of reports of a specific AE for the comparators |
| Feature d | The number of reports of other AEs for the comparators |
| Covariate features | |
| Male | The number of reports of a specific AE for the study drug as male patients |
| Female | The number of reports of a specific AE for the study drug as female patients |
| 0 – 17 years of age | The number of reports of a specific AE for the study drug as patients from 0 to 17 years of age |
| 18 – 64 years of age | The number of reports of a specific AE for the study drugs as patients from 18 to 64 years of age |
| 65 years of age and older | The number of reports of a specific AE for the study drugs as patients aged 65 years and older |
| Serious adverse events | The number of reports of a specific AE for the study drug as serious adverse event |
| Non-serious adverse event | The number of reports of a specific AE for the study drug as non-serious adverse event |
| Spontaneous reporting system | The number of reports of a specific AE for the study drug from spontaneous reporting system |
| Post-marketing surveillance | The number of reports of a specific AE for the study drug from post-marketing surveillance studies |
| Literature | The number of reports of a specific AE for the study drug from literatures |
| Physician | The number of reports of a specific AE for the study drug by physicians |
| Pharmacist | The number of reports of a specific AE for the study drug by pharmacists |
| Nurse | The number of reports of a specific AE for the study drug by nurses |
| Other health professional | The number of reports of a specific AE for the study drug by other health professionals |
| Regional pharmacovigilance center | The number of reports of a specific AE for the study drug from regional pharmacovigilance centers |
| Medical institution | The number of reports of a specific AE for the study drug from medical institutions |
| Manufacturer | The number of reports of a specific AE for the study drug from anufacturers |
| Organ specific feature |  |
| System organ class | System organ class terms assigned to each adverse event in WHO-ART ver.092 |
