# Supplementary material for: Machine Learning for Detection of Safety Signals From Spontaneous Reporting System Data: Example of Nivolumab and Docetaxel
Source: Front Pharmacol. 2021 Jan 14;11:602365. doi: 10.3389/fphar.2020.602365 (PMC7898680; doi:10.3389/fphar.2020.602365)
Supplement: Supplementary file 2 [file table2.docx]

**Table S2.** The predictive performance of machine learning signal detection (MLSD) by adding a rare adverse event feature to the gold standard dataset.

|  | **Optimal threshold** | **Area under curve** | **Sensitivity** | **Specificity** | **Positive predictive value** | **Negative predictive value** |
| --- | --- | --- | --- | --- | --- | --- |
| **Main analysis** | Probability ≥ 0.57 | 0.9643 | 100% | 93% | 95% | 100% |
| **Sensitivity analysis*** |  | 0.9167 | 90% | 93% | 95% | 87% |

*Sensitivity analysis was conducted to identify the robustness of predictive performance of MLSD when adding a rare AE feature to gold standard dataset.
