# Supplementary figures and images for: Machine Learning for Detection of Safety Signals From Spontaneous Reporting System Data: Example of Nivolumab and Docetaxel
Source: Front Pharmacol. 2021 Jan 14;11:602365. doi: 10.3389/fphar.2020.602365 (PMC7898680; doi:10.3389/fphar.2020.602365)

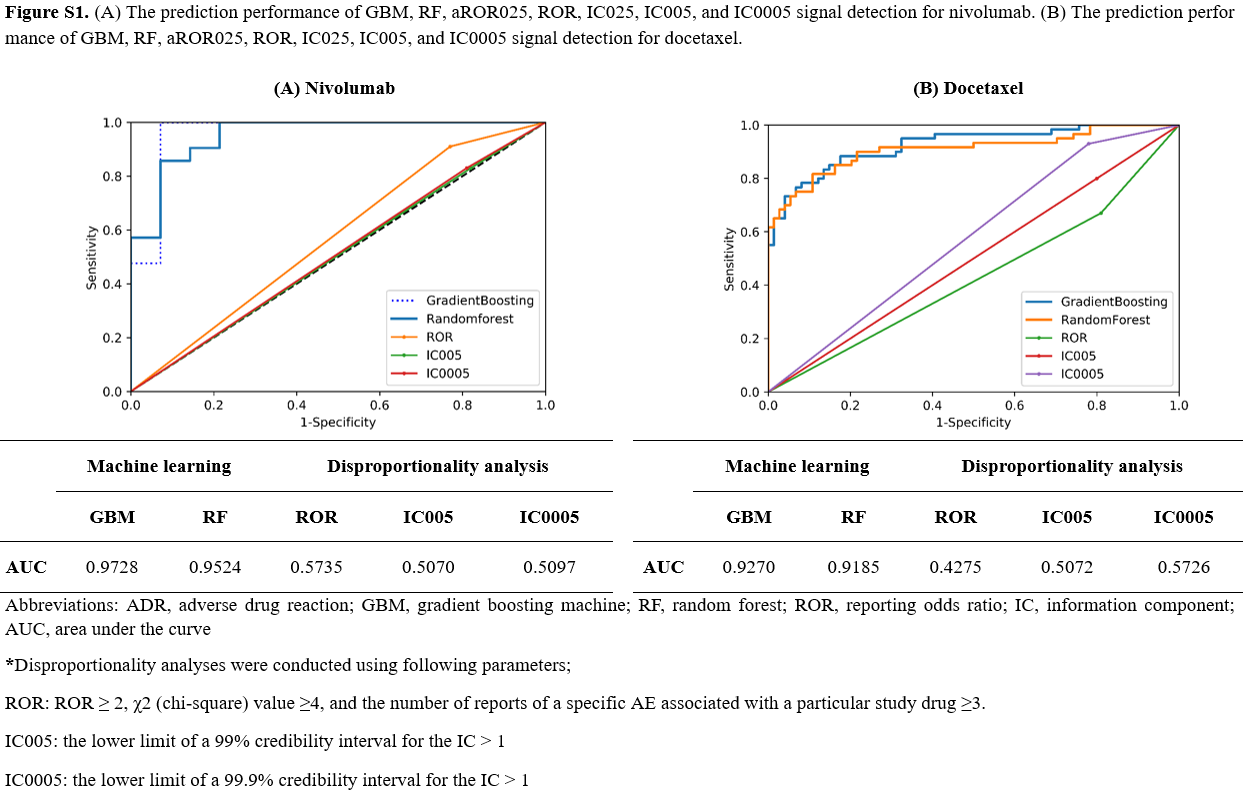

Supplement: Supplementary file 3 [file image1.png]
